# The Impact of the Cost of Travel Time and Feedback Type on Green Travel

**DOI:** 10.3390/bs14070588

**Published:** 2024-07-11

**Authors:** Bowei Zhong, Min Tan, Wen Zhong, Wei Fan

**Affiliations:** 1 Department of Psychology, University of Chinese Academy of Sciences, Beijing 100864, China; zhongbw@psych.ac.cn; 2 CAS Key Laboratory of Behavioral Science, Institute of Psychology, Chinese Academy of Sciences, Beijing 101408, China; 3 Department of Psychology, Hunan Normal University, Changsha 410081, China; mintan2021@163.com (M.T.); zhongwen@csust.edu.cn (W.Z.); 4 Institute of Interdisciplinary Studies, Hunan Normal University, Changsha 410081, China; 5 College of Energy and Power Engineering, Changsha University of Science and Technology, Changsha 410114, China

**Keywords:** green travel, time cost, feedback type, environmental feedback, health feedback

## Abstract

Green travel is a special type of pro-environmental behavior, which requires people to pay a time cost to reduce carbon emissions. This study explored the impact of the cost of travel time and feedback types on green travel. To verify the change of travel choice under different time costs, experiment 1 explored the impact of different costs of travel time on green travel. The results showed that with the increase in time cost, green travel behavior gradually decreased. This suggests that time costs can hinder green travel behavior. To intervene in this negative effect, experiment 2 explored the effects of different types of feedback intervention. The results showed that both environmental and health feedback could only intervene with green travel behavior when the time costs were low. This indicates that health and environmental feedback can intervene in the negative effects of travel time cost, but the cost range of the intervention is limited. This study has implications for promoting green travel behavior.

## 1. Introduction

With the great richness of material life, the ownership rate of private cars is increasing, and the high-carbon phenomenon of private car travel is widespread. Problems such as traffic congestion and air pollution not only affect people’s quality of life but also cause serious harm to sustainable development and health, which have become major issues of social concern [1]. The environmental problems caused by excessive carbon emissions are an urgent problem for ecological remediation. Green travel is one of the most effective ways to reduce air pollution and improve the ecological environment. Green travel originates from the concept of green transportation and aims to guide residents to choose travel methods that save energy, reduce pollution, and are conducive to residents’ health [2], such as public transport, walking, and cycling, rather than using fossil fuel vehicles. Green travel is one of the pro-environmental behaviors [3]. Pro-environmental behavior requires individuals to make certain efforts or pay certain costs to achieve the goal of improving the environment or reducing the impact on the ecological environment [4]. This means that green travel also has this character; that is, to reduce carbon emissions, one needs to pay the cost of time. For example, in most cases, bicycles are slower than private cars and take more time.

Lange et al. [5] developed a series of travel tool choices to measure green travel—the pro-environmental behavior task (PEBT) laboratory paradigm. In each experiment, participants had to decide whether to travel by car or bicycle and generate carbon emissions by turning on lights. Different vehicles have different waiting times, and the participants must wait for the corresponding time after each choice to complete the travel task. PEBT has been used in many studies, and it has been found that as time costs rise, pro-environmental behaviors decline. However, few studies have verified the effectiveness of PEBT in Chinese samples, and it is unclear whether previous findings on the relationship between the cost of travel time and green travel behavior are applicable in Chinese samples. This is because there are cultural differences between countries. For example, compared with Chinese people, British people have more sustainable experiences in travel and a higher ability to overcome obstacles and complete behaviors [6]. It has also been found that Chinese people attach more importance to protecting social interests than to self-enhancement [7]. In addition, previous studies have merely demonstrated a general decline in green travel behavior as time costs increase without highlighting the specific differences between each increment of cost [5]. This could suggest a trend of decline between costs, yet without significant differences. Whether all levels of time costs exert a negative impact on green travel remains unclear. This study aimed to thoroughly understand the impact of different time costs and the differences between the time costs using a Chinese sample. Experiment 1 examined the effects of different time costs on pro-environmental behavior.

Importantly, in real life, the time cost is sometimes inevitable, and people are required to pay their time resources for long-term benefits. How can we encourage people to sacrifice their time for environmental protection? It is well worth investigating how to promote pro-environmental behaviors with a time cost. Humans are learning animals who adjust their choices based on the outcomes of their actions [8]. Feedback evaluation is an important way to guide and optimize behavior [9]. Feedback is the process of providing people with information about their past behavior [10]. Studies have found that feedback can be an effective intervention tool for reducing personal consumption and promoting pro-environmental behavior [11,12,13]. Meta-analyses support the effectiveness of feedback in promoting pro-environmental behaviors [10,11,14]. Evidence from the framework effect suggests that environmental and health feedback frameworks are more likely to promote people’s energy-saving behavior [15,16]. Studies have found that using air pollution and health consequences as health feedback can effectively reduce people’s electricity use [15]. In addition, some studies have found that environmental feedback can reduce individual energy consumption [17]. Providing information on carbon emissions can also lead to sustainable green transportation behavior, and people are willing to adjust their behavior to reduce carbon emissions [18]. This suggests that health and environmental feedback are effective ways to promote pro-environmental behavior.

However, previous studies have not compared whether environmental and health feedback have different effects on pro-environmental behavior. Which is better, environmental feedback or health feedback, or are they both equally effective? In other words, how different feedback types and time costs interact with green travel behavior is still a problem to be solved. Therefore, based on different feedback information, this study explored the impact of time cost and feedback type on pro-environmental behavior (experiment 2). Research has shown that environmental and health feedback can effectively reduce people’s energy consumption behavior [15,17]. Therefore, we hypothesized that both the environmental feedback group and the health feedback group could more effectively engage in pro-environmental behavior with a time cost compared to the control group. Meanwhile, health feedback provides self-related behavioral outcomes, i.e., an individual’s health, and self-serving information is driven by selfish motives [19], which are appropriate for self-serving goals and ease the conflict between self-interest and environmental interest. Therefore, we hypothesized that, compared with environmental feedback, health feedback would have a better effect on green travel behavior with a time cost.

## 2. Experiment 1

### 2.1. Method

#### 2.1.1. Participants

The G-power 3.1 software calculated the number of participants and suggested 19 participants based on 80% statistical power and a moderate effect size [20]. Experiment 1 recruited 40 college students, 21 of whom were female. They were between 18 and 24 years old. All participants had never been involved in a similar experiment, and all rode a bicycle. All participants were given appropriate rewards after the experiment was completed. Two participants exceeded the data by more than 3 standard deviations, and the final data were valid for 38 participants. The experiment was carried out at Hunan Normal University in 2024.

#### 2.1.2. Pro-Environmental Behavior Task (PEBT)

Pro-environmental behavior was measured using PEBT [5]. In this task, participants were required to select between a car and a bicycle, with each option corresponding to a specific waiting time. Prior to making a selection, a travel plan may be presented on the computer screen. For instance, the waiting time for car travel was 5 s, while the waiting time for bicycle travel was 10 s. Thus, if participants selected the car, they were required to wait five seconds to proceed to the subsequent stage of the experiment and generate the carbon emissions produced by driving a car by turning on the lights. Conversely, if they opted for a bicycle, they were obliged to wait ten seconds to complete the journey. The waiting time for a bicycle was always 0, 5, 10, 15, or 20 s longer than the waiting time for a car (5, 10, 15, or 20 s), which made up 24 time combinations.

#### 2.1.3. Cost Manipulation

The time cost was manipulated by the difference between the waiting time of the car and the bike, which is divided into 6 levels, namely 0 s, 5 s, 10 s, 15 s, 20 s, and 30 s.

### 2.2. Procedure

**Figure 1:**
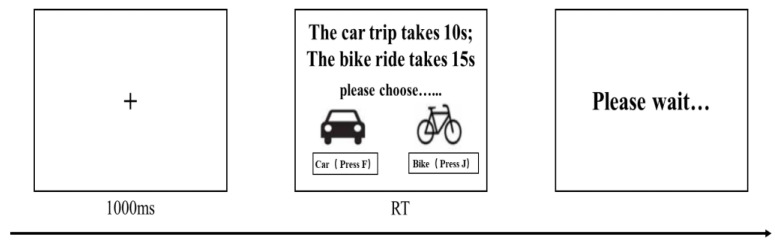
An illustration of a trial.

Before the formal experiment, the participants needed to complete the practice experiment to understand the experiment process. The formal experiment was a series of choices about travel, and the travel time was presented randomly. In the decision-making, the participants were presented with two tools of travel (bicycle and car) to choose from. The participants were instructed to depress a button with their right or left index finger, thereby selecting the vehicle. Subsequently, the participants were required to wait for the allotted time to elapse before commencing the subsequent round. If the car was selected, the lights were illuminated throughout the waiting period. On the other hand, if the bicycle was selected, the lights did not come on (see Figure 1).

### 2.3. Results

**Figure 2:**
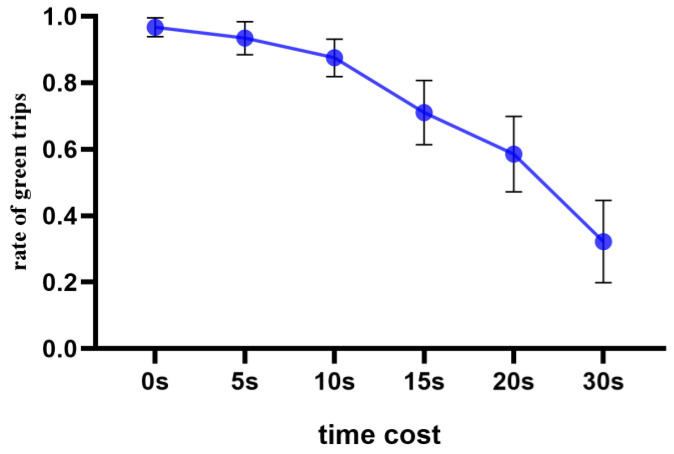
Green travel rate under different time cost conditions.

With time cost as the independent variable and the green travel rate (the total number of options divided by the number of bike options) as the dependent variable, a one-way repeated measure analysis of variance (ANOVA) was performed. The results showed that the main effect of time cost was significant (*F* (5, 33) = 70.98, *p* < 0.001, ƞp2 = 0.66). The post-hoc multiple comparisons showed that the difference between no time cost and a time cost of 5 s was not significant, and other costs were significantly different from no time cost. Other than the difference between the cost of 5 s and 10 s not being significant, the rate of green travel decreased significantly with the increase in cost (see Figure 2).

### 2.4. Discussion

The results of experiment 1 revealed no significant difference between no time cost and 5 s, and no significant difference between time costs of 5 s and 10 s. This was inconsistent with previous studies [5,21]. Studies have found that trials with higher PEBT costs (i.e., a larger difference in waiting time) have higher retest reliability, and trials with a wait time of 10 s may not cause enough variation to provide a reliable measurement [22]. This indicates that when the time cost is low, the retest reliability is also low. In other words, when the time difference is small, the stability and consistency of PEBT are low. However, this does not affect the overall trend of PEBT decreasing with the increase in time cost. Time cost is still a barrier to green travel, which harms green travel. To intervene in the negative impact of time cost on green travel, the intervention effect of feedback type on this negative effect was explored in experiment 2.

## 3. Experiment 2

### 3.1. Method

#### 3.1.1. Participants

The G-power 3.1 software calculated the number of participants and suggested 93 participants based on 80% statistical power and a moderate effect size [20]. Experiment 2 recruited 106 college students, 66 of whom were female. They were between 18 and 24 years old. All of the participants had never been involved in a similar experiment, and all rode a bicycle. All participants were given appropriate rewards after the experiment was completed. The data for one participant exceeded 3 standard deviations, and the final data were valid for 105 participants. Among them, 34 were in the control group, 38 were in the health feedback group, and 33 were in the environmental feedback group. The dependent variable tasks used in the two experiments, all information collected from the respondents, and the references used for the manipulation of the independent variables and the dependent variable tasks can be found in Table 1. Cost manipulation and dependent variable tasks are consistent with experiment 1. The experiment was carried out at Hunan Normal University in 2024.

#### 3.1.2. Feedback Manipulation

Feedback was the outcome that resulted from the choices made by the participants. The health feedback showed the amount of pollution and prevalence rate caused by their choice [15], while the environmental feedback showed the amount of carbon dioxide emissions caused by the behavioral choice [17]. Specifically, in the health feedback group, when the participant chose to drive a 5-s car, the following health feedback was presented: your travel has produced 616 mg of pollutant emissions and increased the morbidity of residents. If the participant chose a bicycle, the health feedback was: your travel has reduced emissions by 616 mg and reduced the morbidity of residents. Driving time determines the number of pollutants emitted and CO_2_ emissions. Similarly, in the environmental feedback group, if participants chose to drive a 5-s car, the following feedback was presented: your travel has increased CO_2_ emissions by 140 g. If a bicycle was selected, the feedback told them their travel had reduced CO_2_ emissions by 140 g. Data on carbon dioxide and pollutants emitted by vehicles were obtained from the Emission Limits and Measurement Methods for Light Vehicle Pollutants (China Phase 5), which came into effect in 2018. The control group were presented no feedback about the chosen outcome.

### 3.2. Procedure

**Figure 3:**
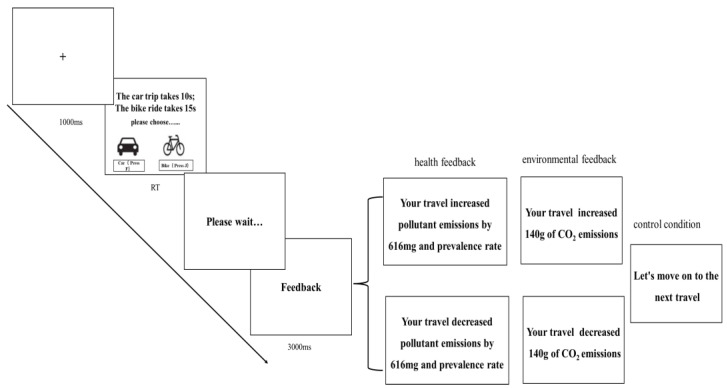
An illustration of a trial in experiment 2.

The experimental procedure for the control group was roughly the same as that of experiment 1, while the feedback group increased the presentation of feedback information based on experiment 1. When the participants came to the lab, they were randomly assigned to one of three groups. Participants first completed the practice experiment to understand the procedure and then began the formal experiment. In the formal experiment, participants completed the PEBT and were presented with a feedback message when the waiting period was over. The feedback presented to the control group was to move on to the next trip. The feedback presented to the health feedback group was based on the increase or decrease in pollutant emissions and the morbidity associated with the vehicle selected by the participant at the decision interface. The environmental feedback group was presented feedback on whether their travel increased or decreased carbon dioxide emissions. After receiving the feedback, participants proceeded to the next trial (see Figure 3).

### 3.3. Results

**Figure 4:**
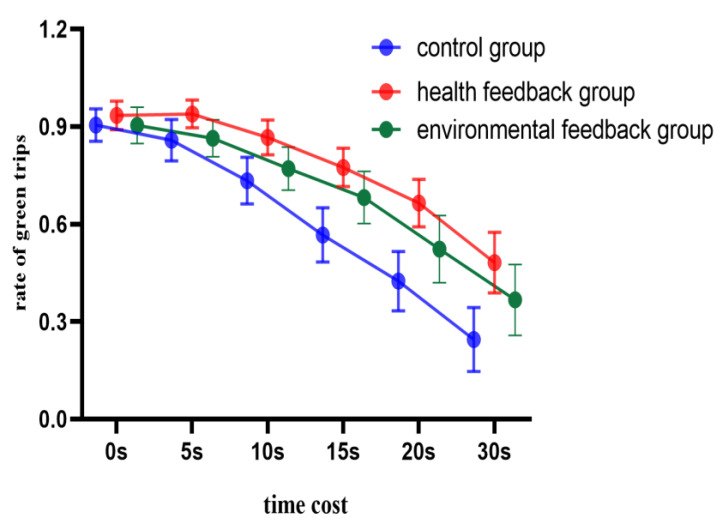
Green travel rate of three groups under different time cost conditions.

With time cost and feedback type as the independent variables and green travel rate as the dependent variable, a two-way repeated measures ANOVA was performed. The results showed the main effect of time cost was significant (*F* (5, 98) = 195.77, *p* < 0.001, ƞp2 = 0.66), and the proportion of green travel decreased significantly with the increase in time cost. The main effect of feedback was significant (*F* (2, 102) = 8.50, *p* < 0.001, ƞp2 = 0.14). The proportion of green travel in the health feedback group (*M* = 0.78) was significantly higher than that in the control group (*M* = 0.62) and environmental feedback group (*M* = 0.69) (*p* < 0.001). There was no significant difference between the control group and the environmental feedback group (*p* = 0.334), and no significant difference between the environmental and the health feedback groups (*p* = 0.054). Importantly, the interaction was significant (*F* (5, 98) = 2.96, *p* = 0.022, ƞp2 = 0.06). The simple effects analysis found significant differences across all costs in the control group (*F* (5, 98) =25.52, *p* < 0.001, ƞp2 = 0.58). In the environmental feedback group, there was no significant difference between no time cost and a time cost of 5 s, but significant differences between other time costs (*F* (5, 98) = 18.09, *p* < 0.001, ƞp2 = 0.48). In the health feedback group, there were no significant differences between no time cost and time costs of 5 and 10 s, while there were significant differences between other time costs (*F* (5, 98) = 15.05, *p* < 0.001, ƞp2 = 0.43) (see Figure 4).

### 3.4. Discussion

Experiment 2 explored the impact of feedback type and time cost on green travel. The results showed that, compared with the control group, the environmental feedback group can effectively improve the green travel proportion at a time cost of 5 s, while the health feedback group can improve the green travel proportion at a time cost of 10 s. This suggests that health feedback is more effective than environmental feedback in improving green travel behaviors. However, when the time cost is equal to or higher than 15 s, neither type of feedback has a significant intervention effect, indicating that the effect of feedback is limited. These results are elaborated on in detail in the general discussion.

## 4. General Discussion

### 4.1. The Impact of Time Cost on Green Travel

Experiment 1 found that the proportion of green travel between a time cost of 5 s, no time cost, and a time cost of 10 s was similar, which was somewhat inconsistent with previous studies. Experiment 2 found that with the increase in time cost, the proportion of green travel decreased significantly, which was consistent with previous studies. The results of the two experiments are different when the time cost is small, which may be because the retest reliability of PEBT is low when the time cost is low [21], and it is easily affected by other factors. Importantly, both experiments found a downward trend in the proportion of green trips as the time cost rises, which is consistent with previous research, which found that for every minute increase in travel time required to use public transport, the odds of driving increased by about 2.2–3.0% [23]. People weigh the relative value of individual benefits and environmental costs, and they are less likely to participate in pro-environmental activities that require more behavioral costs [24], leading to more pro-environmental behavior as individual costs decrease [25]. The rational choice approach holds that people are selfish actors who make rational choices based on costs and benefits to maximize the utility of behavior [26]. The Random Regret Minimization (RRM) model analyzes travel choice behavior on the basis of utility maximization, capturing the traveler’s choice behavior based on minimizing the perceived regret decision criterion. People make travel choices in a way that minimizes expected regret, i.e., the chosen option should be characterized by the highest efficiency, the best plan, and the greatest satisfaction [27]. When environmental protection does not require people to pay costs (i.e., no cost), taking environmental protection behavior does not involve costs and can bring environmental benefits, and pro-environmental behavior has become a utility choice that minimizes expected regret. However, when pro-environmental behavior requires people to pay a cost, that is, pro-environmental behavior involves a conflict between personal interests and long-term interests, people are less likely to act according to normative considerations because they threaten the realization of hedonic and selfish goals [28]. This means that the utility and satisfaction of pro-environmental behavior have begun to decline. When the time cost is higher, individuals need to pay more personal benefits, making the dilemma or conflict in environmental decision-making stronger and the threat of achieving self-interested goals greater. Thus, people’s satisfaction with and use of green travel behavior are greatly reduced. In other words, when people perceive that choosing a car is better for them than choosing a bicycle, the travel choice that minimizes expected regret is the non-environmental protection behavior, not the pro-environmental behavior.

### 4.2. The Impact of Time Cost and Feedback Type on the Green Travel

To explore the intervention effect of environmental and health feedback on time cost, experiment 2 manipulated different feedback information in PEBT. The results show that environmental feedback can only improve green travel behavior that has a time cost of 5 s and does not affect other time costs. This is inconsistent with previous studies. Environmental feedback activates altruistic motivation. When an action benefits others and the environment, people have a strong intention to be environmentally friendly, even at a high cost [17]. This can be caused by the gap between intention and actual behavior. Environmental attitudes can only predict low-cost pro-environmental behavior [25,29]. Although people may claim that they are willing to protect the environment at a high cost, the actual behavior pays a real cost, which means the positive environmental attitude cannot be translated into actual behavior. The gap between intention or attitude and actual behavior often occurs in pro-environmental behavior [30,31,32]. Meanwhile, the cost needs to be paid now, and the environmental impact of carbon dioxide emissions caused by lighting cannot be significantly changed immediately but will occur in the future. Distant future consequences lack urgency and may be psychologically underestimated, making it difficult for people to adopt pro-environmental options [33]. This limits the effectiveness of environmental feedback, making people willing to make only a very low effort to deal with the environmental consequences of significant changes in the future. This suggests that the impact of environmental feedback on actual behavior is limited to behavior with very low costs. The results also found that health feedback can improve green travel behavior at time costs of 5 and 10 s. Health feedback provides information related to self-health and belongs to the self-interest motive [34], which does not pose a threat to the self-interest goal and can ease the dilemma in pro-environmental decision-making.

Not entirely consistent with the hypothesis, health feedback is not useful for all costs; it is only useful for low time costs, not for time costs longer than 10 s. This suggests that health feedback only alleviates self-interest threats that cost less than 10 s. Research has found that unhealthy information about the self may be perceived as a threat to self-worth and is often resisted [35]. When reminded of the unhealthy message of the behavior, people often feel defensive and reject the information because acknowledging the behavior undermines a person’s positive self-image [36]. This suggests that providing unhealthy information about behavior may pose a threat to self-worth or self-image. When an individual chooses a car, the health feedback presents unhealthy information about the behavior, which leads to a threat to self-worth and thus weakens the effect of the self-interest motive on the dilemma. Self-threat can weaken the role of self-interest, but the dilemmas involved in low-cost behavior are not so strong that there is no need to involve very strong self-interest. Even if the effect of self-interest motive is diminished, it can still play a role. However, the higher the time cost, the stronger the self-interest motive that is needed to alleviate the dilemma conflict because the threat to the self-interest goal is greater. In other words, strong self-interest motivation is needed to alleviate the high cost, which is a scenario that self-interest weakened by self-threat cannot cope with. Therefore, health feedback can only work for green travel behavior with low time costs.

According to the mindsponge-mindspongeconomics system, external information carries different values and is processed by a filter-based information processing system [37]. It is assumed that the new information is similar to the core value, in which case it can be integrated into the mindset and then behaviors can be adopted that are consistent with the value of the new information [38]. Compared with the altruistic value carried by environmental feedback, the value carried by health feedback is self-serving. Core value usually involves preference, cultural value, convenience, subjective cost–benefit judgment, and other components. Among them, the subjective cost–benefit changes dynamically according to the situation. Health feedback with self-interest is more easily matched to core values than environmental feedback with altruism because self-related information has higher utility and is more likely to conform to an individual’s preferences. This suggests that health feedback is more likely to be accepted by core values than environmental feedback, which in turn prompts people to adopt more pro-environmental behaviors. However, the benefit of health feedback is very limited, only having an effect in the case of the time cost of 10 s. In both the moderate and high-cost scenarios, the effects of health feedback and environmental feedback are similar. This indicates that the core value may change under moderate and high costs, which is mainly caused by subjective cost–benefit judgment. Under the time cost scenario of more than 10 s, the perceived cost is too high, which greatly reduces the benefit of health feedback, and then the pro-environmental behaviors are similar to those when receiving environmental feedback. The mindsponge-mindspongeconomics system offers another possible explanation for the difference between environmental feedback and health feedback.

### 4.3. Theoretical and Practical Contributions

This study has some theoretical implications. The RRM model holds that people can choose the travel behavior with the highest utility and satisfaction to minimize expected regret [27]. When environmental behavior has no cost and environmental benefits, it becomes a higher utility choice that minimizes expected regret. As the cost increases, the greater the price that will be paid for choosing environmental behaviors, the stronger the threat to self-interested goals, and the progressively lower the utility and satisfaction of environmental behaviors that do not achieve the goal of minimizing the expected regret. The mindsponge-mindspongeconomics system holds that when new information matches core values, individuals may take actions consistent with the new information. Among them, subjective cost–benefit judgment is one of the components of the core value [38]. Health feedback has greater subjective utility than environmental feedback, is more compatible with core values, and can promote low-cost green travel behavior. In the case of moderate and high costs, the role of health information cannot offset the weight of cost; that is, the cost is too high, so the utility of health feedback is reduced, and then it cannot promote green travel behavior with moderate and high costs.

This study also has some practical implications. Health feedback can intervene in or promote green travel behavior more than environmental feedback. Non-profit organizations or public service advertising can highlight the health information relevant to environmental protection, such as “environmental protection is good for your health”. However, marketers and environmental organizations should recognize that the impact of health feedback as an intervention is limited to low-cost green travel behavior. We cannot expect this single strategy to change green travel behavior at all costs.

### 4.4. Limitations and Future Directions

First of all, all participants were students and the sample size was small, which resulted in a limited number of groups applicable to the research findings. Future studies may consider expanding the sample scope to further verify the external validity of the results. Meanwhile, for the sample of young adults used in this study, there may be significant differences in pro-environmental behaviors among different age groups. Future studies could expand the sample to include participants of different ages. This will help us better understand how age affects green travel behavior and reveal differences between different age groups. Secondly, the two experiments in this study obtained different results on the verification of PEBT. Future studies may consider using cross-cultural methods to explore the stability of PEBT. Finally, this study only concerned the role of environmental feedback and health feedback and did not consider other types of feedback. Some studies have found that both emotional feedback [13] and personalized feedback [39] are effective in promoting pro-environmental behavior. In the future, it may be considered whether these two kinds of feedback are more effective than environmental feedback and health feedback as interventions.

## 5. Conclusions

In summary, our research reveals the impact of time costs and feedback type on green travel. The results show that with the increase in time cost, green travel behavior decreases gradually. Time costs may threaten the achievement of self-serving goals and reduce the utility of environmental protection behavior. Importantly, this study uses health feedback and environmental feedback as interventions to encourage people to make time sacrifices for the sake of environmental protection. The manner in which distinct types of feedback influence green travel behavior varies. Environmental feedback may stimulate individual altruistic motivation, while health feedback may trigger direct personal interest. The results show that environmental feedback can improve green travel behavior with a time cost of 5 s, and health feedback can promote green travel behavior with a time cost of fewer than 10 s. This indicates that health feedback can promote people’s green travel behavior more than environmental feedback. In other words, self-related health information can better motivate people to devote time to environmental protection, which can promote low-cost green travel behavior. Our findings support the possibility of using feedback interventions to promote behavior change. Providing health feedback can effectively motivate people to adopt more environmentally friendly methods of travel.

## Figures and Tables

**Table 1:** Information adopted and collected in the experiment.

Experiment	Task	Feedback	Demographic Variables	References
Experiment 1	PEBT		Age; Sex Name	Lange, F. et al., (2018) [5]
Experiment 2	PEBT	Health and environmental feedback	Age; Sex Name	Lange, F. et al., (2018) [5]; Asensio, O. I., & Delmas, M. A. (2015) [15]; Dogan, E. et al., (2014) [17]

## Data Availability

The data supporting the findings of this study are available from the corresponding author upon reasonable request.
